# Bees increase crop yield in an alleged pollinator-independent almond variety

**DOI:** 10.1038/s41598-020-59995-0

**Published:** 2020-02-21

**Authors:** Agustin Sáez, Marcelo A. Aizen, Sandra Medici, Matias Viel, Ethel Villalobos, Pedro Negri

**Affiliations:** 1 Grupo de Ecología de la Polinización, INIBIOMA, CONICET-Universidad Nacional del Comahue, San Carlos de Bariloche, Rio Negro, Argentina; 2 Centro de Investigación en Abejas Sociales (CIAS) (IIPROSAM-CONICET), Universidad Nacional de Mar del Plata (UNMdP), Funes 3350, CP 7600 Mar del Plata, Argentina; 3 Beeflow Inc. - Smart Pollination Services, Los Angeles, CA USA; 4 Department of Plant and Environmental Sciences, University of Hawaii at Manoa, Honolulu, HI 96822 USA

**Keywords:** Plant domestication, Agroecology

## Abstract

Wild pollinators are declining and the number of managed honey bee colonies is growing slower than agricultural demands for pollination. Because of these contrasting trends in pollinator demand and availability, breeding programs for many pollinator-dependent crops have focused on reducing the need for pollinators. Although numerous crop varieties are now available in the market with the label of pollinator-independent, the real dependence of these varieties on pollinators is mostly unknown. We evaluated the hypothesis of pollinator independence in the *Independence* almond variety, the fastest growing variety in California that is the main almond production region in the world. In this presumed pollinator-independent variety, we measured the effect of honey bees on fruit set, yield, and kernel nutritional quality at tree level. Fruit set was 60% higher in bee-pollinated than bee-isolated trees, which translated into a 20% increase in kernel yield. Despite its effect on almond production, there was no evidence that bee visitation affected almond nutritional quality. Based on these results, we recommend the use of bees, whether they are wild or managed, to maximize yield even in self-fertile almond varieties.

## Introduction

Dependence on animal pollination is increasing in global agriculture^1^. In 2005, the total estimated value of pollination worldwide was about $172 billion and accounted for nearly 10% of the world’s agricultural crop production consumed by humans^2^. However, this percentage is increasing year after year as the area cultivated with pollinator-dependent crops is continuously expanding^3^. Unfortunately, wild pollinators are declining whereas managed honey bees are growing slower than agricultural demands for pollination^4,5^. Because of these uneven trends in pollinator demand and availability, breeding programs for many pollinator-dependent crops are targeted to reducing the need of biotic transfer of pollen for ovule fertilization, and thus for seed and fruit production. Many varieties of several typically pollinator-dependent crops are now available in the market with the label of “self-fertile” and sold as pollinator-independent. However, the extent to which these varieties depend on pollinators for either yield quantity or quality under field conditions is unknown.

Almond (*Prunus dulcis*) production in California, USA, clearly illustrates this trend. California produces more than 80% of world’s almonds, and the area devoted to this crop is continuously growing^6,7^. For decades, almond varieties cultivated in California have been self-incompatible and growers have relied almost exclusively on managed honey bees (*Apis mellifera*) for pollen transfer and effective pollination^8,9^. Because of almond’s high pollinator-dependence and high-market value, more than a million honey bee colonies are moved to California from all over the U.S. every season, representing the largest man-driven pollination event in the world^10,11^. However, the number of honey bee colonies has been dwindling in the U.S. over the last decades^12^. Although new evidence is showing that the number of colonies has stabilized during the last years^13^, beekeepers are still dealing with several health problems affecting honey bees. These health problems can be related in part to long-distance colony movement, which facilitates the spread of pathogens^14^. In parallel, the almond-cultivated area has expanded and demands for almond pollination services have increased^6,10,11^. Because of these opposing trends, the average rental rate for single honey bee colony has increased from ~$70 in 1995 to an average of ~$180 in 2018^10,15^. After accounting for inflation, costs of renting colonies are now ~60% higher than two decades ago^7,10,15^.

Under this scenario, the self-fertile almond variety named *Independence* (Independence^R^ (Alm-21 cv.) Patent no. 20295) was developed, becoming increasingly popular among Californian growers^16^. This was meant to be a massive breakthrough for almond industry, because this new variety was advertised as bee-independent due to self-fertility and its presumed high capacity for autonomous self-pollination^7,17,18^. Thus, cultivation of this variety would, in principle, benefit growers by removing the costs of renting bee colonies, among other advantages. In fact, the area cultivated with self-fertile *Independence* increased exponentially since its release in 2008^16^. However, despite claims of bee-independence, there is an information gap on the real pollinator dependence of this fast-growing almond variety.

Given the profound impact that replacements of self-incompatible by self-compatible crop varieties can exert on production, food quality and beekeeping financial well-being, an accurate evaluation of pollinator dependence on much promoted bee-independent varieties appears as a straightforward priority. Here, we measured the effect of bees on fruit set, kernel yield, and kernel nutritional quality on the “self-fertile” *Independence* almond variety. If this variety were fully self-fertile and capable of autonomous self-pollination, we should observe a similar fruit set, and kernel yield and quality in trees visited as in those not visited by bees.

**Figure 1:**
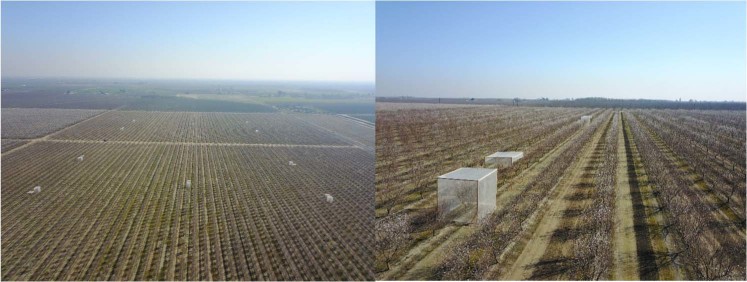
Aerial images of the experimental setup. Left image, almond fields with experimental trees. Right image, a closer look of the treatment “blocks”, consisting of a group of three neighboring trees, each one randomly assigned one the three different pollination treatments.

Thirty almond trees of the *Independence* variety were used in our experiment. Ten trees were entirely covered with a fine mesh normally used by growers to isolate citrus trees from bees (henceforth “isolation treatment”). Another ten trees were partially covered with a mesh above, without interfering with bee visitation to flowers but to control for possible effects of irradiation and frost (henceforth “control treatment”). The remaining ten trees were kept open to bees (henceforth “open treatment”; see Fig. 1 and Supplementary Information S1). Honey bee colonies were placed at a stocking rate of five colonies/ha surrounding the experimental study fields, which is a standard stocking rate for self-incompatible varieties^19^.

During flowering, we quantified visitation rate of bees to flowers in open and control trees. We recorded only flower visitors that contacted anthers and/or stigma. After flowering, we quantified initial and final fruit set (i.e., proportion of flowers setting a fruit) in all thirty trees. Finally, during harvest, we quantified almond yield (i.e., the entire kernel production at tree level) and nutritional quality. Because the main components in almond used to value its nutritional quality are fats, and particularly the proportion of oleic to linoleic fatty acids, here we analyzed the oleic to linoleic fatty acid ratio of dry kernels (see M&M).

## Results

### Flower visitation

Managed honey bees accounted for all flower visits to the self-compatible *Independence* almond variety. We did not find evidence that bee visitation differed between open-pollinated and mesh-control trees (in *log* scale, *β* = −0.29, SE = 0.31, *Z* = −0.92, *P* = 0.35), with an observed mean (±SE) of 0.05 ± 0.010 and 0.04 ± 0.008 visits · flower^−1^ · 5 min^−1^, respectively. Taking into account that almond flowers remain open for about 4–6 days and were visited during ~5 h each day, this visitation rate represents approx. 12–18 visits during a flower’s lifespan.

### Initial and final fruit set

**Figure 2:**
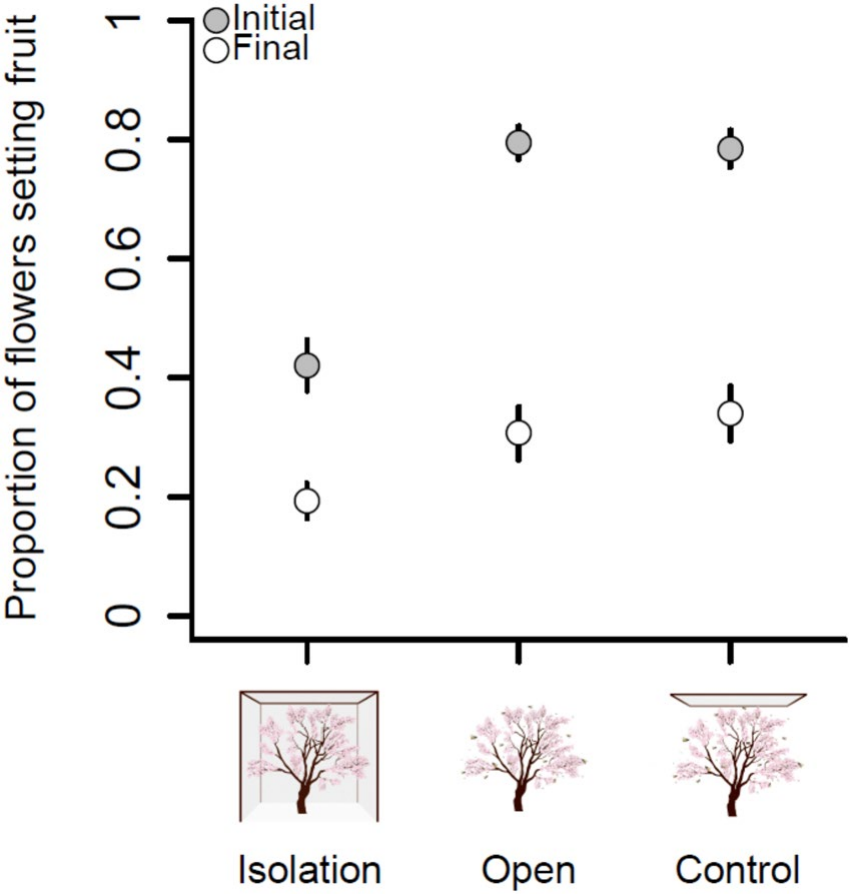
Initial and final fruit set per pollination treatment. Effects of the pollination treatment (isolation, open and control) on initial (gray) and final (white) fruit set. Points represent mean values and bars represent two standard errors.

Bee visitation had a positive effect on the likelihood of a flower setting a fruit. Initial fruit set (about three weeks after the end of flowering) was ~90% higher in bee-pollinated than bee-isolated trees (in *logit* scale, *β* = 1.70, SE = 0.17, *Z* = 9.65, *P* < 0.001; and *β* = 1.62, *SE* = 0.17, *Z* = 9.23, *P* < 0.001; for comparisons between the isolation and open, and isolation and control treatments, respectively). Also, initial fruit set did not differ significantly between open-pollinated and mesh-control trees (in *logit* scale, *β* = −0.08, SE = 0.18, *Z* = −0.44, *P* = 0.89). Initial fruit set in isolated trees was, on average (±SE), 0.42 ± 0.02 fruits/flower, and in open-pollinated and mesh-control trees was 0.79 ± 0.01 and 0.78 ± 0.01 fruits/flower, respectively (Fig. 2, gray points). Final fruit set (i.e., before harvesting) was ~60% higher in bee-pollinated than bee-isolated trees (in *logit* scale, *β* = 0.63, SE = 0.18, *Z* = 3.37, *P* = 0.002; and *β* = 0.75, SE = 0.18, *Z* = 4.03, *P* < 0.001; for comparisons between the isolation and open, and isolation and control treatments, respectively). Also, final fruit set of open-pollinated and mesh-control trees was not statistically different (in *logit* scale, *β* = 0.12, SE = 0.18, *Z* = 0.66, *P* = 0.78). Final fruit set in isolated trees was, on average (±SE), 0.19 ± 0.01 fruits/flowers, and in open-pollinated and mesh-control trees was 0.30 ± 0.02 and 0.33 ± 0.02 fruits/flower, respectively (Fig. 2, white points). Even though many fruits abort early on during development, increases in fruit set in bee-pollinated trees persist until fruit maturity.

### Kernel yield

**Figure 3:**
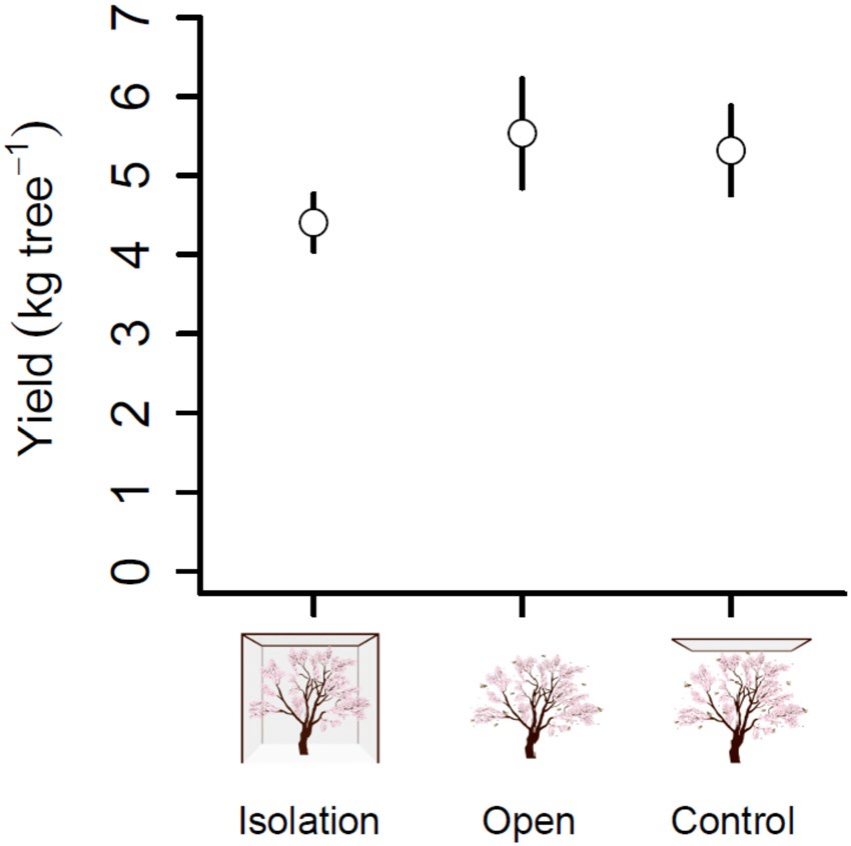
Quantitative yield per treatment. Effects of the pollination treatment (isolation, open and control) on mass production of kernels at tree level. Points represent mean values and bars represent two standard errors.

Increases in fruit set translated into increases in kernel yield in bee-pollinated trees. Kernel yield (i.e., estimated as total fruit mass produced per tree times the proportion of the fruit weight represented by the kernel; see Supplementary Information S3) was ~20% higher in bee-pollinated than bee-isolated trees (*β* = 1.04, SE = 0.39, *t* = 2.65, *P* = 0.01; and *β* = 0.82, SE = 0.33, *t* = 2.45, *P* = 0.02; for comparisons between the isolation and open, and isolation and control treatments, respectively). Mean (±SE) kernel yield in isolated trees was 4.49 ± 0.18 kg · tree^−1^, whereas kernel yield in open-pollinated and mesh-control trees was 5.53 ± 0.39 and 5.31 ± 0.33 kg · tree^−1^, respectively (Fig. 3), with no statistical differences between these two latter treatments (*β* = 0.003, SE = 0.006, *t* = 0.63, *P* = 0.802).

### Almond nutritional quality

Despite effects on almond quantity, there was no evidence that bee visitation affected almond quality. Specifically, mean (±SE) ratio of oleic to linoleic acid in kernels from isolated trees was 2.63 ± 0.04, a similar ratio to that found in kernels from trees pollinated by bees (*β* = −0.11, SE = 0.06, *t* = −1.87, *P* = 0.16; and *β* = −0.05, SE = 0.06, *t* = −0.89, *P* = 0.64; for comparisons between the isolation and open, and isolation and control treatments, respectively). Mean (±SE) ratio of oleic to linoleic acid in kernels from open-pollinated and mesh-control trees was 2.52 ± 0.06 and 2.58 ± 0.06, respectively, with no statistical differences between these two treatments (*β* = 0.05, SE = 0.06, *t* = 0.92, *P* = 0.62). Therefore, whereas bee pollination affected almond seed quantity, it did not seem to affect seed quality.

## Discussion

Self-fertile almond varieties are becoming increasingly popular among growers and, consequently, their cultivated area is increasing at a high rate^16^. However, studies estimating true pollinator dependence in tree crops are still scarce. This may relate to the complexity of conducting this estimation at the level of entire trees, like we did here. Most studies assessing pollinator dependence in multi-flowered plants compare fruit or seed set between isolated and open-pollinated individual flowers or inflorescences, which can lead to overestimation of pollinator dependence due to the possibility of resource translocation^20^. Here, we evaluated the hypothesis of pollinator independence in the *Independence* almond variety, which is the fastest growing variety in California, in turn, the most important almond production area globally^16^. When bee visitation to *Independence* trees was curtailed, trees did in fact produce many fruits. Nevertheless, honey bee visits to almond flowers of this variety increased yield substantially. This result implies that growers will be experiencing lower crop yields if bees are not present in areas where this variety is cultivated, and thus, the hypothesis of complete pollinator independence should be rejected.

Until recently, all almond cultivars were self-incompatible, characterized by a gametophytic system controlled by a *S*-locus^21^. For this reason, almond orchards include a mix of different interfertile varieties, honey bees being a required input to ensure cross pollination. Approximately, 80% of the U.S. honey bee colonies have been used for almond pollination in California during 2018^22^. In turn, pollination services for the almond industry represent one third of the U.S. beekeeping incomes, and many beekeepers rely to a great extent on the almond pollination market for profit^23^. As a consequence, almond growers and beekeepers have developed a strong inter-dependency.

Given the potential economic benefits for almond growers resulting from the disruption of their partnership with beekeepers, autonomous self-pollination and self-fertility have been priority target traits in almond breeding programs, traits which not only avoid costs of renting colonies but also facilitate management though the cultivation of monovarietal plantations^18,24–26^. Although most of the new varieties decreased dependency from bees, they did not seem to achieve total pollinator independence. They did, however, allow growers to decrease stocking rates of managed honey bee colonies per cultivated area and to overcome production costs involved in inter-varietal mixing^23^. The *Independence* variety was expected to achieve the ultimate goal of not requiring bee visitation for maximizing yield and income, and was advertised accordingly^17,18^. However, here we provide evidence that this variety could be truly self-fertile but not totally pollinator independent, probably because of incomplete autonomous self-pollination.

The impact of misleading information on the true pollinator dependency of the *Independence* variety could be detrimental for almond growers and beekeepers in terms of potential profits, labor losses, and social conflicts. First, misinformation on decreased honey bee usage could lead to a reduction in profits for almond growers, because non-bee visited trees will translate into yields lower than maximum potential. After considering kernel yield per tree, the differential price related to kernel class size and colony renting costs (see Supplementary Information S4), bee pollination still translated into differences of about 20% and 10% in gross and net profit (i.e., without and with consideration of renting colonies), respectively. These economic benefits were observed in young trees (i.e., below their yield potential), and thus, the benefits could be even higher in full production plantations. Therefore, bee pollination affected positively the yield and economic profit in this self-fertile almond variety. Second, if *Independence* continues growing and replacing self-incompatible varieties, many beekeepers may lose one of their key annual incomes, threatening their financial well-being. This could lead, eventually, to the loss of one the most important incentives for maintaining beekeeping industry in the U.S. and the narrowing of a work source for beekeepers. Third, honey bees from colonies rented by neighboring growers would visit *Independence* plantations, which will dilute bee density in fields cultivated with the self-incompatible almond varieties. This could represent an advantage for *Independence* almond growers, because the presence of these honey bees will increase their yields at no cost. At the same time, this could cause yield drops for those growers that actually paid for the pollination service. This scenario could generate social conflicts between neighboring growers, particularly being aware of the casual benefits received by their neighbors.

One critical question regarding crop varieties is their nutritional quality. Brittain *et al*.^27^ showed that self-pollinated almond trees produce almonds with lower nutritional quality, in terms of their fat composition, than those produced by cross pollination. However, we did not detect a sizable effect of bee pollination on almond nutritional quality in this study. Differences in results could be attributed to the variety studied, because Brittan *et al*.^27^ worked with *Nonpareil*, the most commonly planted self-incompatible variety in the U.S., and we did it with self-compatible *Independence*. In terms of fat composition quality, oleic to linoleic ratio estimated in the present work for *Independence* was, on average, 2.5, which is within the ranges (i.e., 1.79–3.79) estimated for all the varieties cultivated in California^28^. Therefore, any effect of bees on production in this self-compatible crop variety seems to occur through increases in yield quantity rather than in nutritional quality.

Concerns about the decline of wild pollinators, increasing cost of colonies, and stability in food production caused the introduction of self-compatibility and self-pollination traits as key factors in many plant breeding programs^5,24–26^. Although nowadays there are many varieties available in the market with the “self-fertile” and “pollinator independent” labels, the real dependence on pollinators was not rigorously estimated in most cases. Misleading information from nursery companies on pollinator dependency levels can trigger cascading effects among growers and beekeepers like we describe above. Under this context, reliable and precise information is crucial for profit maximization and socio-economic stability. Breeding programs are clearly reducing pollination dependence for ensuring food production, but bees are still needed for full fertilization. Based on our results and their implications, we highly recommend to almond growers the use of bees, whether they are wild or managed, to maximize yields, even in self-fertile almond varieties.

## Materials and Methods

### Experimental site

The experiment was carried out from February to August 2018. The site was located in Kings County (36° 21.698′ N, 119° 42.263′ W) California, USA. We selected young almond trees for this experiment, because kernel quantity and quality of whole trees can be measured, and accumulated resources are limited in smaller trees^29,30^, which make any test on pollinator dependence more conservative. Two 20-ha plots planted with 50–50% of *Nonpareil - Independence* tree varieties were used to perform the experiment. Sampled trees were approximately 4-m tall and had been harvested twice before this study. Honey bee colonies were placed at a stocking rate of five colonies/ha surrounding the experimental study site and were part of the commercial pollination management system used by the grower.

### Experimental design

To evaluate honey bee effects on fruit set, and kernel yield quantity and quality at tree level, we randomly selected 30 experimental almond trees of *Independence* variety on two plots (i.e., 15 trees per plot). To each tree, we assigned one of the following treatments: (*i*) isolation (i.e., trees were isolated from bees by covering them with a fine mesh 10% Crystal, Green-tek, Sultana, California 93666), (*ii*) open (i.e., trees were kept open to bees), and (*iii*) control (i.e., trees covered with mesh above and along a fringe on the sides, without interfering with bee visitation, to control for effects of the net on attenuating irradiation and frost) (see Fig. 1 in main text and Supplementary Information S1). Each treatment (isolation, open, control) was replicated in 10 trees. Treatments were set in “blocks”, consisting of a group of three neighboring trees, each one randomly assigned one the three different pollination treatments (see Fig. 1 and Supplementary Information S1). In the isolation and control treatments, trees were mesh-covered before blooming started and the mesh immediately removed after bloom was over.

### Data collection and analysis

#### Bee visitation

In open-pollinated and mesh-control trees, we performed 43 pollinator censuses, respectively. The censuses consisted in recording the number of flowers visited by each visiting pollinator to a group of flowers for a period of 5 min (i.e., no. visits · flower^−1^ · 5 min^−1^), totaling ~7 h of observation. Whenever possible, censuses of open and control trees were made simultaneously. The number of observed flowers per census was, on average (±SE), 52 ± 2.54. Each census conducted on each tree, involved a different randomly selected group of flowers. Each tree was surveyed between 11:00 and 17:00. During pollinator censuses, we recorded only flower visitors that contacted anthers and/or stigma. Furthermore, the enclosure of the isolated trees was checked throughout the flowering period to certify the absence of bees.

We evaluated the effect of pollination treatment (open and control) on visitation frequency to flowers with a generalized linear mixed-effects model. Data analysis was carried out using the *lmer* function from the *lme4* package^31^ of the R software^32^. Because response variables were counts (i.e., visits), we used a Poisson error distribution with a *log* link function. We included number of flowers observed in each census as an *offset* (i.e. a fixed predictor known in advance to influence insect visitation)^33^. The pollination treatment was included in the model as fixed effect and each tree nested within a block as a random effect, allowing the intercept to vary among blocks/trees.

#### Fruit set

We tagged five branches in each experimental tree (totaling 150 experimental branches) in which we counted the number of open flowers between the tag and the end of the branch. After bloom, we counted the number of developing fruits to estimate initial fruit set (i.e. fruits/flower ratio). Considering that during “*June drop*” trees lose many fruits, we made a second measurement of fruits retained by trees right before harvest to estimate final fruit set.

We evaluated the effects of the pollination treatment (i.e., isolation, open, and control) on the initial and final probability of a flower setting a fruit with a generalized linear mixed-effects model. Data analysis was carried out using the *lmer* function from the *lme4* package^31^ of the R software^32^. Because the response variable is binary (i.e., a flower setting or not a fruit), the model assumed a binomial error distribution with a *logit* link function. Pollination treatment was considered as a fixed effect and each tree nested within a block as a random effect.

#### Fruit weight and yield

At fruit maturity, all fallen fruits were collected, after shaking the trees, and sun-dried for 5 days. From each tree, we randomly sampled 70 fruits, totaling 2100 fruits (i.e., 70 fruits/ tree × 10 trees/ treatment × 3 treatments). From each individual fruit, we weighed pericarp, endocarp, and kernel (see Supplementary Information S3). Then, we weighed the entire fruit production of each experimental tree with a hand digital scale. To estimate kernel production for each tree, we multiplied total fruit weight times the average proportion of a fruit’s weight represented by the kernel (see Supplementary Information S3).

We used an ANOVA model to estimate the effect of pollination treatment (isolation, open, and control) on kernel production at tree level. Data analysis was carried out using the *gls* function from the R software^32^. Because our data did not comply with the assumption of homogeneous variance, we re-ran the analysis using a heterogeneous variance model with the *varIdent* function, which increased model fit (lower AIC) and provided compliance with model assumptions. We made multiple comparison of means with Tukey post-hoc test using the *glht* function from the *multcomp* package^34^.

### Kernel nutritional quality

We randomly collected ~50 fruits from each tree to estimate kernel nutritional quality. Samples were stored in paper bags and kept at room temperature until laboratory analyses. Fats are the main nutritional component in almond^28^. In particular, clinical studies have shown benefits to human health of mono-unsaturated fats present in almonds^35^. Here we described and analyzed almond’s fatty acid portion. In particular, we estimated the oleic to linoleic acid ratio, which is the most common measure to determine almond nutritional quality^27^. Details of analytical methods employed for estimations of fatty acid composition are provided in the Supplementary Information S2.

We estimated effects of the pollination treatment (isolation, open, and control) on almond’s oleic and linoleic acid content, and oleic to linoleic ratio at tree level by means of an ANOVA model. Data analysis was carried out using the *lm* function from the R software^32^. We made multiple comparison of means with Tukey post-hoc tests using the *glht* function from the *multcomp* package^34^.

## Supplementary information

Supplementary information.

## Data Availability

The datasets generated for this study are available on request to the corresponding author.
