# Supplementary material for: Bees increase crop yield in an alleged pollinator-independent almond variety
Source: Sci Rep. 2020 Feb 21;10:3177. doi: 10.1038/s41598-020-59995-0 (PMC7035345; doi:10.1038/s41598-020-59995-0)
Supplement: Supplementary file 1 — Supplementary information. [file 41598_2020_59995_MOESM1_ESM.pdf]

Supplementary Information

Bees increase crop yield in an alleged pollinator-independent almond variety

Agustin Sáez, Marcelo A. Aizen, Sandra Medici, Matias Viel, Ethel Villalobos, & Pedro Negri

Supplementary S1. Visual support of experimental design……………………. Pag 2

Supplementary S2. Quantification of fatty acids ……………..………………. Pag 3

Supplementary S3. Fruit components………………………………………… Pag 5

Supplementary S4. Yield profits……………………………………………..... Pag 7

Supplementary S1. Visual support of experimental design.

Image S1. A. From left to right: isolation, open and control (see M&M) treatments in almond trees (*Prunus dulcis* L.) of the study *Independence* variety in full bloom. Ten trees were used per pollination treatment, totaling 30 experimental trees. **B.** Full view of isolated and mesh control treatments in almond trees of the *Independence* variety. **C.** Initial fruit set estimation in one of the tagged branches. Incipient fruits still preserving the sepals, anthers and stigma of the flower. We tagged five branches per tree for fruit-set estimation, totaling 150 branches (i.e., 30 trees x 5 branches). **D.** Fruits harvested at tree level before being weighted. **E.** Different parts form the almond fruit. From left to right: pericarp, endocarp and kernel. **F.** Honey bee (*Apis mellifera* L.) collecting pollen in an almond flower during the experimental assay.

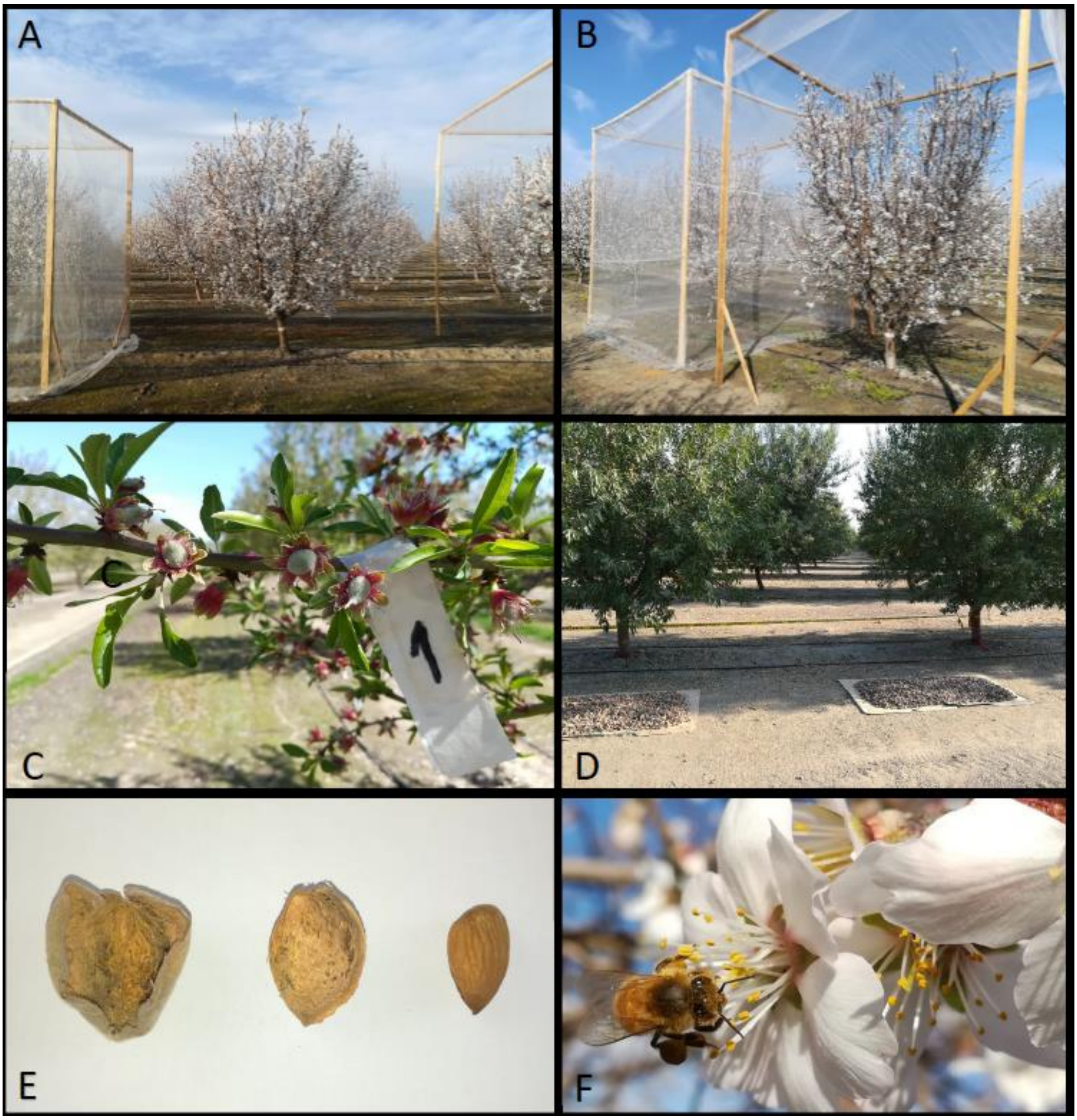

Supplementary S2. Quantification of fatty acids

Since the main nutritional component in almond are fats [1], we analyzed the fatty acid portion of the almond kernel. Particularly, we estimated the oleic and linoleic acid content, and the oleic to linoleic ratio. Samples from each tree were processed, and their fat was extracted following a modified Folch [2] technique as follows:

1. *Extraction.* Almonds randomly collected from sampled trees (see M&M "Kernel nutritional quality" in main text) were finely crushed. Then, we weighed 1 ± 0.1 g of the finely crushed almonds and added 4 ml of the extraction solution (chloroform: methanol 2:1). Samples were stirred in a vortex and let stand for 30 min; then they were sonicated 60 minutes and filtered. The filtrate was incubated in an over at 44 ± 2 ° C for 24 hours. The crystalized fat was re-suspended in 3 ml of pure chloroform (purity $\geq$ 98%). The resulting solution was then filtered using a syringe with a 0.45 µm filter and poured into a 4 ml vial. After the chloroform had evaporated, the vials were closed and stored at 4 ºC.
2. *Esterification of fatty acids*. One milliliter of acid methanol (1% HCl in methanol) was added, and then the vials were taken to the stove at 44 ± 0.1ºC for 24 hours. The next day, we added a 1ml of n-hexane (purity > = 98%) and shacked the sample in a vortex. In a 2 ml vial, we added to the sample 950 µl of dichloromethane (purity 99.9%) and 50 µl of esterified hexane (upper phase of the 4 ml vial). The sample was then run in a gas chromatograph (HP 6890) with a FID detector, using a column Thermo 7HG-G033-10 FAME ZB and the following chromatographic conditions: (*i*) a constant flow of 2 ml/min, (*ii*) an injection volume of 1 µl, and (*iii*) a temperature of the detector of 275°C. The standard used was a mixture of 37 fatty acid esters (SIGMA®) Accustandard brand.
3. *Quantification.* Presence and retention times of 37 fatty acids were verified using the FAME (SIGMA®) standard run along each batch. To quantify the fatty acids in each sample, we located the peaks corresponding to each fatty acid according to their retention times and recorded the area of each peak. Values were reported as a percentage, so that the total peak area across all fatty acids for each sample added 100%.

Origin of the reagents used:

1. Clorophorm, analytic grade, Ciccarelli
2. Methanol, HPLC grade, UVE, Slot UZ190H2006
3. ClNa, analytic grade, Biopack
4. HCl, analytic grade, Ciccarelli
5. n-Hexane, pesticide grade, Merck, slot I0957971
6. Estandard for HPLC: FAME FAMQ-005 (37 components), Accustandard, slot 217101392

References S2

1. Sathe, S. K., Seeram, N. P., Kshirsagar, H. H., Heber, D. & Lapsley, K. A. Fatty acid composition of California grown almonds. *J. Food Sci.* **73**, C607–C614 (2008).
2. Lees, M. & Sloane, G. H. A simple method foe the isolation and purification of total lipides from animal tissues. *J. Biol. Chem.* **226**, 497–509 (1957).

Supplementary S3. Fruit components

To estimate kernel production at tree level, we multiplied the total fruit weight produced per tree by the weight proportion that the kernel represented in the samples (i.e., total fruit weight x kernel weight proportion). To this end, we randomly sampled 70 fruits from each experimental tree from which we measured pericarp, endocarp and kernel weight separately. We examined components of fruit weight of a total of 2,100 fruits (70 fruits/tree x 10 trees/treatment x 3 treatment).

We evaluated the effects of the pollination treatment (Isolation, Open, and Control) on pericarp, endocarp and kernel weight with general linear mixed-effects models. Data analysis was carried out using the *lme* function from the *nlme* package [3 4] of the R software [5]. Because of the response variables were continuous (i.e.,weight), we assumed a Gaussian error distribution in the three models. In all cases, the pollination treatment was included in the model as a fixed effect and plot and tree within plots as random effects, allowing the intercept to vary among plots and trees.

Treatment affected individual fruit weight. Fruits from isolated trees weighed (mean ± SE) 4.74 ± 0.06g, and were ~13% heavier than those from bee-pollinated trees (β = -0.61, *SE* = 0.16, *Z* = -3.87, $P < 0.001$; and β = -0.66, *SE* = 0.16, *Z* = -4.13, $P < 0.001$; for the open and control treatments, respectively). Also, there were no significant differences in fruit weight between Open and Control treatments (β = -0.05, *SE* = 0.16, *Z* = -0.32, *P* = 0.94), that weighed 4.13 ± 0.07g and 4.07 ± 0.07g, respectively. Therefore, non-bee visited almond trees produced fewer fruits (see main text) but somewhat heavier.

Overall fruit weight differences were not identical/equivalent for each constituting part. First, pericarps from isolated trees were ~18 % heavier than those from bee-pollinated trees. The pericarp of fruits from the isolated trees weighted 2.62 ± 0.03g, while pericarps from open and mesh-control trees weighted 2.17 ± 0.03g and 2.14 ± 0.03g, respectively (Figure S3). Second, endocarps from isolated trees were ~12% heavier than those from trees pollinated by bees. The fruit endocarp from isolated trees weighted 0.61 ± 0.008g, while endocarps from open and mesh-control weighted 0.54 ± 0.007g and 0.53 ± 0.005g, respectively (Figure S3). Third, kernels produced by isolated trees were 8% heavier than those by trees open to bee pollination. Kernels from isolated trees weighted 1.50 ± 0.02g, while kernels from open and shaded trees weighted 1.40 ± 0.01g and 1.38 ± 0.01g, respectively (Figure S3). Thus, in relative terms, differences in kernel weight between bee and non-bee visited trees were much smaller than the differences found between the other fruit components.

As we showed here, the fruit weight proportion represented by the kernel is different in isolated trees than in those pollinated by bees. In isolated trees, the weight of the kernel represented, on average (± SE), 31 ± 0.2% of the total fruit weight, while in open and control trees the weight of the kernels represented in both cases 34 ± 0.2%. For this reason, to estimate kernel production at the tree level we multiplied total fruit weight per tree times 0.31 for isolated trees, and times 0.34 for open and control trees.

Figure S3. Effects of pollination treatments (i.e., isolation, open and control) on the weight of the different parts of the almond fruit (i.e., pericarp, endocarp, and kernel). Thick bars represent mean values, while the thin black bars two standard errors.

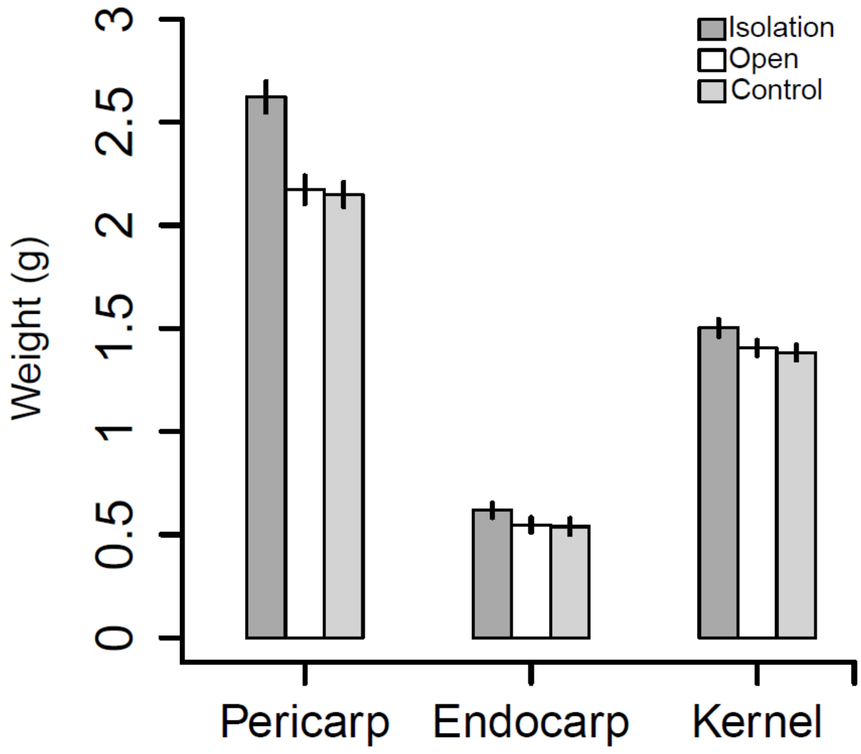

References S3

3. Bates, D. nlme: Linear and Nonlinear Mixed Effects Models. *R Packag. version* 31–128 (2007).

4. Bates, D., Mächler, M., Bolker, B. & Walker, S. Fitting linear mixed-effects models using lme4. *arXiv Prepr. arXiv1406.5823* (2014).

5. Cran.R-Project.org. R Software - v3.4.4. https://cran.r-project.org/bin/windows/base/old/3.4.4/

6. Connell, J. H., Index, A. D., Prunus, W., California, S. U. & Webb, M. D. A. Pollination of Almonds : Practices and Problems. *Horttechnology* 10, 116–119 (2000).

Supplementary S4. Yield profits.

To calculate the profit associated with almond production, we first categorized all sampled kernels by class size (also called “ounce count”). This classification reflects the number of kernels per ounce, where fewer kernels per once mean higher quality (larger) kernels. Second, we estimated the proportion of kernels in each class size or marketable size category (Table S4) for each tree and multiplied these proportions by total kernel production per tree. This allowed us to estimate weight of kernels in each size category produced by a tree. Third, the profit per tree for each treatment was translated into US dollars by multiplying the estimated weight of kernels in each kernel size category by the 2018 Harris Woolf's pricing list of the *Independence* variety for each kernel size category, and then adding these values up (see Table S4). Finally, we calculated profit at the plot level (per ha) by multiplying the average profit per tree times the average number of trees planted per ha (i.e., ~336 trees/ha). The rental cost of honey bee colony was included in the final calculation of the profit per ha.

Kernel production quality in terms of size did not differ strongly between non-bee and bee-pollinated trees. While non-bee pollinated trees produced 94% of the kernels from the first three sizes category (i.e., high quality), bee pollinated trees produced 83%. After accounting for the differential price related to kernel size category, the revenue per tree was ~20% lower in isolated trees than in those trees pollinated by bees (Table S4). Non-bee-pollinated trees generated a revenue $25.04 USD, while bee-pollinated trees ~ $30 USD. Assuming 336 trees · ha^{-1} at the field level, young (~ 4 years) non-bee plantations would produce ~$8.400 USD · ha^{-1}, while bee-pollinated plantations would produce ~$10.000 USD · ha^{-1}.

Adding the cost of five colonies per ha (on average, 180 USD per colony), the cost-benefit balance is still positive, even in young, low-productive trees like we studied here. Also, although here we could not estimate the optimal range of bee visits needed to maximize yield, two colonies per acre, as used in self-incompatible varieties [6], probably provide more bees visits than needed to maximize yields in this self-compatible almond variety.

Table S4. Production quality classification and profit from the tree and plantation levels for each pollination treatment. “Avg.” denotes averages; “Class size” indicates the number of kernels per ounce (i.e. 28.38 gr); “Price” indicates the USD price for one kilogram (kg) of almond kernels using the *Independence* variety pricing list from Harris Woolf 2017. Production was calculated in kilograms (kg), and profits are in USD. Totals per ha were calculated assuming a tree density of 336 trees · ha^{-1}. In this study, experimental trees were in their fourth year of production.

		**Avg. % of kernels**			**Avg. tree production (kg)**			**Avg. revenue ($)**		
**Class size**	**Price ($)**	**Isolation**	**Open**	**Control**	**Isolation**	**Open**	**Control**	**Isolation**	**Open**	**Control**
**20AOL**	**5.77**	43.6	22.2	23.8	1.916	1.219	1.260	11.055	7.036	7.269
**20/22**	**5.77**	30.8	32.7	27.3	1.353	1.796	1.445	7.809	10.364	8.338
**22/25**	**5.66**	17	29.4	30.6	0.747	1.615	1.620	4.228	9.140	9.167
**25/27**	**5.55**	3.9	8.8	8.7	0.171	0.483	0.460	0.951	2.683	2.556
**27/30**	**5.44**	2.6	4	5.2	0.114	0.220	0.275	0.622	1.195	1.497
**30/32**	**5.33**	0.7	0.8	1.6	0.031	0.044	0.085	0.164	0.234	0.451
**32/34**	**5.22**	0.4	0.6	0.3	0.018	0.033	0.016	0.092	0.172	0.083
**34AOS**	**4.88**	0.6	1.2	2	0.026	0.066	0.106	0.129	0.322	0.517
				**Total per tree**	4.377	5.476	5.267	25.049	31.146	29.878
				**Total per ha**	1470.5	1840.0	1769.6	8416.4	10464.9	10038.9
							**Colonies ($)**	0	900	900
							**Profit ($)**	8416.4	9564.9	9138.9
